# ‘Sadly I think we are sort of still quite white, middle-class really’ – Inequities in access to bereavement support: Findings from a mixed methods study

**DOI:** 10.1177/02692163221133665

**Published:** 2022-11-06

**Authors:** Lucy E Selman, Eileen Sutton, Renata Medeiros Mirra, Tracey Stone, Emma Gilbert, Yansie Rolston, Karl Murray, Mirella Longo, Kathy Seddon, Alison Penny, Catriona R Mayland, Donna Wakefield, Anthony Byrne, Emily Harrop

**Affiliations:** 1 Palliative and End of Life Care Research Group, Population Health Sciences, Bristol Medical School, Bristol, UK; 2 Cardiff School of Dentistry, Cardiff University, Cardiff, UK; 3 Ubele Initiative, London, UK; 4 Marie Curie Research Centre, Cardiff University, Cardiff, UK; 5 Wales Cancer Research Centre, Cardiff, UK; 6 National Bereavement Alliance, London, UK; 7 Department of Oncology and Metabolism, University of Sheffield, Sheffield, UK; 8 North Tees and Hartlepool NHS Foundation Trust, Stockton-on-Tees, UK

**Keywords:** Health equity, grief, pandemics, bereavement, coronavirus infections, bereavement services, palliative care

## Abstract

**Background:**

Voluntary and community sector bereavement services are central to bereavement support in the UK.

**Aim:**

To determine service providers’ perspectives on access to their support before and during the COVID-19 pandemic.

**Design:**

Mixed methods study using an explanatory sequential design: (1) Cross-sectional online survey of UK bereavement services; (2) Qualitative interviews with staff and volunteers at selected services.

**Settings/participants:**

147 services participated in the survey; 24 interviews were conducted across 14 services.

**Results:**

67.3% of services reported there were groups with unmet needs not accessing their services before the pandemic; most frequently people from minoritised ethnic communities (49%), sexual minority groups (26.5%), deprived areas (24.5%) and men (23.8%). Compared with before the pandemic, 3.4% of services were seeing more people from minoritised ethnic groups, while 6.1% were seeing fewer. 25.2% of services did not collect ethnicity data. Qualitative findings demonstrated the disproportionate impact of the pandemic on minoritised ethnic communities, including disruption to care/mourning practices, and the need for culturally appropriate support. During the pandemic outreach activities were sometimes deprioritised; however, increased collaboration was also reported. Online provision improved access but excluded some. Positive interventions to increase equity included collecting client demographic data; improving outreach, language accessibility and staff representation; supporting other professionals to provide bereavement support; local collaboration and co-production.

**Conclusions:**

Service providers report inequities in access to bereavement support. Attention needs to be paid to identifying, assessing and meeting unmet needs for appropriate bereavement support. Identified positive interventions can inform service provision and research.

**What is already known about the topic?**

- There are known inequities in who receives formal bereavement support, with, among others, people from minoritised ethnic communities, sexual minority groups and people with lower socio-economic status known to experience barriers to access.
- The COVID-19 pandemic had a disproportionate impact in the UK, with higher mortality and bereavement rates in minoritised ethnic minority communities and groups with lower socio-economic status.

**What this paper adds**

- 67.3% of voluntary and community sector bereavement services in the UK reported that there were population groups with unmet support needs which experienced barriers to accessing their service before the pandemic, with minoritised ethnic groups most frequently recognised in this regard.
- Despite the disproportionate and multi-dimensional impact of the pandemic on minoritised ethnic communities, for the majority of bereavement services in the UK, the proportion of clients from these communities did not increase and in some cases decreased during the pandemic.
- Positive interventions to increase equity included monitoring client characteristics to identify gaps; improving outreach, language accessibility and staff representation; supporting other professionals in the community to provide bereavement support; local collaboration and co-production of services to ensure appropriateness and inclusivity.

**Implications for practice, theory or policy**

- More needs to be done to tackle inequity in access to bereavement support – and many service providers both recognise this and are ready to implement changes to widen access to their support.
- Prioritising equity means identifying, assessing and meeting unmet needs in bereaved communities, adapting services and outreach to ensure inclusivity and working in partnership with communities and community-based organisations.
- Study findings can help inform efforts to widen access and reduce inequities.

## Background

A direct cause of approximately 6 million deaths to date, COVID-19 has brought bereavement support centre-stage as a core element of health and social care provision.^1^ Public health approaches to bereavement recommend a tiered approach based on level of need.^2,3^ Tier 1 includes universal access to information on grief and available support, recognising that many bereaved people cope without formal intervention, drawing on their existing social networks. Tier 2 includes individual and group-based support for those with moderate needs, who have been shown to benefit from increased social support and opportunities for reflection, emotional expression and restorative activities. Tier 3 specialist mental health and psychological support is effective for those with high-level needs and at risk of prolonged grief disorder and should be targeted at those identified as meeting these criteria.^2,4,5^

In the UK, voluntary and community sector bereavement services play a crucial role in providing tier 2 and 3 bereavement support. Bereavement sector policies^6–10^ mandate equitability and fair access, yet there is evidence that certain population groups are less likely to proactively seek out and access professional care and support – even when needed and wanted – and are more likely to feel uncomfortable asking for help.^11,12^ A systematic review identified barriers to accessing bereavement support among LGBTQ+ communities as well as additional stressors, including discrimination, homophobia, disenfranchisement, historical illegality and higher rates of social isolation.^13^ Another systematic review highlighted access barriers among minoritised ethnic communities: experiences of institutional racism (including in healthcare), a lack of awareness of bereavement support (often due to poor information provision by professionals^14^ and a lack of outreach by services), the type and/or format of support being culturally or individually inappropriate, and stigma regarding mental health within some minoritised communities.^15^ A scoping review examining inequity following expected death described how specific groups of bereaved people may be disadvantaged and disenfranchised in multiple ways, due to varied dimensions of their structural vulnerability, with gender, class and age acting as additional, intersecting axes of inequity.^16^ The review found that bereavement itself can constitute a form of social inequity, exposing grieving individuals to policy, processes, systems and networks that function in disenfranchising ways, for example via an apparent esteem of processes that promote ‘productivity’ and ‘stoicism’.^17^ All three reviews found a lack of evidence regarding the experiences and needs of structurally vulnerable populations, their receptivity to and engagement with bereavement support, and how bereavement services can best support them.^14–16^

These findings are particularly concerning given the disproportionate impact of the COVID-19 pandemic on people with lower socio-economic status^18^ and minoritised ethnic groups,^19,20^ reflecting underlying social, structural and economic inequalities,^21^ and of an inequitable response by palliative care providers.^22^ We aimed to contribute to the evidence base for equitable bereavement support by describing access to voluntary and community sector bereavement support in the UK, as reported by these organisations, and exploring bereavement service providers’ views and experiences of providing support during the COVID-19 pandemic.

## Methods

### Design

A pragmatic, explanatory sequential mixed methods study^23^ comprising:

- An online cross-sectional open survey of voluntary and community sector bereavement services in the UK, disseminated via national organisations, networks and social media (March–May 2021).
- Qualitative semi-structured telephone interviews with staff/volunteers at selected bereavement services (June–December 2021) which aimed to expand on the survey findings.

Here we present findings related to the equitability of bereavement support, using the Checklist for Reporting Results of Internet E-Surveys^24^ in reporting. This work is part of a larger research study which also examined experiences of bereavement during the pandemic in the UK.^14,25–27^

*Setting and population*: Voluntary and community sectors bereavement services in the UK.

### Sampling

*Survey*: Convenience sample of voluntary and community sector bereavement services.

*Qualitative interviews*: We aimed to purposively sample 8 to 12 bereavement support organisations from the 147 organisations who completed the online survey. Sampling aimed to capture diverse organisations and experiences during the pandemic, considering: organisation size; geographical area; type of support provided; support for specific groups (e.g. minoritised ethnic communities, children and young people); reported challenges and innovations during the pandemic. In addition, we included two UK social media communities providing support to people bereaved during the pandemic, as these were an important source of support which was not captured in the survey.

### Recruitment

*Survey*: A link to a JISC^28^ survey was disseminated to voluntary and community sector bereavement services, via emails from the research group and national bereavement organisations and associations, national stakeholder webinars, and social media, and posted to the study website (covidbereavement.com). We asked one representative from each organisation to participate, consulting with colleagues as needed.

*Qualitative interviews*: Potential participants (one at each selected organisation) were sent an invitation, information sheet and consent form. After the initial interview, the team decided whether or not to recruit additional staff/volunteers from the organisation via snowball sampling, considering the data collected and the size and nature of the organisation. Snowball sampling aimed to capture additional perspectives, for example manager/team lead in addition to bereavement counsellor. All participants gave written consent.

### Data collection

*Survey*: The survey (Supplemental File 1) comprised non-randomised open and closed questions exploring the impact of the pandemic on bereavement services and their response, including closed and open questions on access, with additional information specifically requested about clients from minoritised ethnic communities. Survey items were based on the literature and initial scoping of the pandemic’s impact,^29,30^ with input (including testing) from an expert advisory group of researchers, clinicians, bereavement support practitioners and people with experience of bereavement.

*Qualitative interviews*: Telephone interviews were conducted using a semi-structured topic guide (Supplemental File 2; adapted for online services), developed as above. Interviews were conducted by ES (*n* = 21), EG (*n* = 2) and LES (*n* = 1), experienced qualitative researchers. Fieldnotes were taken to inform sampling, data collection and analysis.

### Analysis

*Survey*: All data are categorical. Graphical summaries, including pie charts, bar charts and stacked bar charts, were used to describe all variables. Logistic regressions were performed to investigate which factors (area served, type of organisation, client group/age, whether restricted by cause of death or age of deceased) might be associated with reporting they were not reaching specific community groups with unmet needs; the proportion of clients from minoritised ethnic groups and whether the organisation collected ethnicity data. All analyses were performed by RMM using R (version 4.1.1, R Core Team, 2021), implemented in R-Studio (www.r-studio.com). Free-text data were analysed using thematic analysis^31,32^ in NVivo12^33^ by TS, discussed with LES and ES and refined.

*Qualitative interviews*: Interviews were transcribed verbatim and checked for accuracy prior to thematic analysis^31,32^ in NVivo12^33^. Analysis used a combination of deductive and inductive coding strategies and was conducted concurrently with data collection, allowing insights from earlier interviews to inform those conducted subsequently. ES, LES and TS read and independently coded a sub-set of interview transcripts and developed a coding framework which ES applied to the dataset. ES and LES met regularly to discuss the development and revision of key themes and sub-themes,^34^ drawing out differences, similarities and patterns in the data.

Quantitative and qualitative findings were triangulated and integrated into a narrative, with the latter used to explain and add richness to quantitative findings.^23^ All quotations are anonymised, with pseudonyms used in data extracts.

### Ethical approval

Ethical approval for the study was granted by the University of Bristol, Faculty of Health Sciences Ethics Committee (Ref: 114304 20/12/2020).

## Results

### Participants

**Figure 1.**
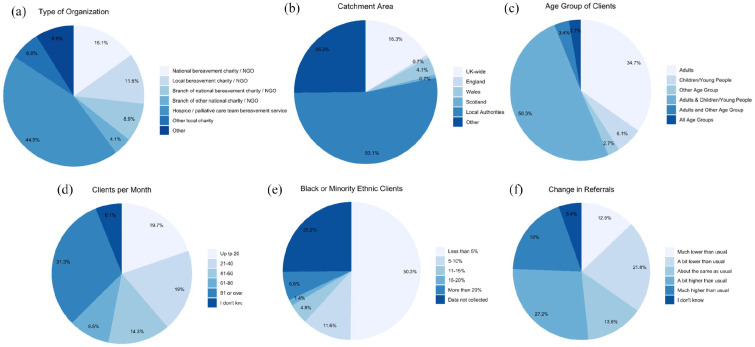
Characteristics of services and change in referrals (*n* = 147, except for type of Organisation *n* = 146).

*Survey*: 147 bereavement services from across UK regions participated (Figure 1). As this was an open survey the response rate is not known (see *Limitations*). Two participants completed the survey twice; their first and second responses were merged. Two services provided two responses; the second response from each was excluded. 44.5% were hospice or palliative care services (including services part-funded by the NHS); 15.1% national bereavement charities or non-governmental organisations (NGOs); 11.6% local bereavement charities/NGOs; 8.9% branch of a national bereavement charity/NGO; 4.1% branch of other national charity/NGOs; 6.8% other local charities/NGOs; 8.9% other (e.g. council-commissioned service, local collaborative partnership, community-led initiative or community interest company). 68% provided support following all causes of death whereas 32% were focused on specific causes of death such as terminal illness. Services provided the following levels of support pre-pandemic: Information on grief and sign-posting to other services (*n* = 122, 83.0%); Group meeting of peers (people with similar experiences but no one is trained) (*n* = 74, 50.3%); Group meeting facilitated by someone with training (*n* = 114, 77.6%); One-to-one support (e.g. individual or family counselling by someone with training) (*n* = 128, 87.1%); Specialist intervention involving mental health services, psychological support services or specialist counselling/psychotherapy (*n* = 65, 44.2%).

*Qualitative interviews*: Twenty-four interviews with staff and volunteers from 14 organisations were conducted (Table 1). Two services provided targeted support for specific minoritised ethnic communities (Muslim and African Caribbean). Fourteen survey respondents were initially sampled and approached via email; 3 did not respond and 11 participated. A further 12 participants were contacted via snowball sampling; 2 did not respond and 10 participated. Two potential participants coordinating social media communities were contacted via email and both participated; an additional volunteer was recruited via snowball sampling. Interviews lasted 25 to 77 min (mean 46 mins).

**Table 1.** Details of organisations and interview participants.

Organisation ID	Size of Org	Type of service	Group(s) supported	Bereavement services provided (pre-Covid unless stated)	Geographical area	Interviewee role
Org A	Regional	Hospice	Bereavement support for children and young people (and those caring for them) and adults following all causes of death	Information on grief and sign-posting to other services; group meetings of peers; group meetings facilitated by someone with training; one-to-one support e.g. counselling Other services: Pre-death support Immediate post-death support Remembrance services Drop-in support	South East	**A1:** Manager **A2:** Director
Org B	Small	Bereavement charity mainly supporting minoritised group (Muslim community)	Bereavement support for children and young people (and those caring for them) and adults following all causes of death	Information on grief and sign-posting to other services; group meetings facilitated by someone with training; one-to-one support e.g. counselling Other services: Pre-death support Drop-in support	UK wide	**B1:** Director **B2:** Volunteer
Org C	Branch of National Organisation	Bereavement charity	Bereavement support for children and young people (and those caring for them) and adults following all causes of death	Information on grief and sign-posting to other services; group meetings of peers; group meetings facilitated by someone with training; one-to-one support e.g. counselling Other services: Pre-death support	West Midlands	**C1:** Regional Manager **C2:** Volunteer
Org D	Small	Bereavement NGO supporting minoritised group (African Caribbean community)	Bereaved adults following all causes of death	Information on grief and sign-posting to other services; group meetings facilitated by someone with training; one-to-one support e.g. counselling	UK Wide	**D1:** Director
Org E	Regional	Hospice/palliative care service part-funded by NHS, Regional Bereavement Network	Bereavement support for children and young people (and those caring for them) and adults following death from a life-limiting illness	Information on grief and sign-posting to other services; group meetings of peers; group meetings facilitated by someone with training; one-to-one support e.g. counselling; specialist intervention involving mental health services/specialist counselling Other services: Pre-death support Immediate post-death support Condolence letters Home visits Remembrance services Support with funerals Walking group Gardening group	Scotland	**E1:** Team Lead **E2:** Volunteer
Org F	Small, Regional	Bereavement charity, Regional Bereavement Network	Bereavement support for children and young people (and those caring for them) following all causes of death	Information on grief and sign-posting to other services; group meetings of peers; group meetings facilitated by someone with training Other services: Pre-death support Immediate post-death support Home visits Drop-in support Online community Activity days	Scotland	**F1:** Coordinator
Org J	Regional	Charity	Bereavement support for adults following death from a life-limiting illness	Group meetings facilitated by someone with training; one-to-one support e.g. counselling Other services: Individual and group therapy for people facing death and those close to them	South West	**J1:** Clinical Lead **J2:** Senior Therapist
Org K	Regional	Hospice/palliative care service part-funded by NHS	Bereavement support for children and young people (and those caring for them) and adults following all causes of death	Information on grief and sign-posting to other services; group meetings of peers; group meetings facilitated by someone with training; one-to-one support e.g. counselling Other services: Pre-death support Immediate post-death support Home visits Remembrance services	North East	**K1:** Senior Practitioner **K2:** Volunteer
Org P	Branch of National Organisation	Palliative care charity	Bereavement support for adults known to the palliative care service following death from a life-limiting illness	Information on grief and sign-posting to other services; group meetings of peers; group meetings facilitated by someone with training; one-to-one support e.g. counselling; specialist intervention involving mental health services/specialist counselling Other services: Pre-death support Immediate post-death support Condolence letters Home visits Remembrance services	South East	**P2:** Specialist Counsellor
Org N	Small, Regional	Hospice	Bereavement support for children and young people (and those caring for them) and adults following all causes of death	Information on grief and sign-posting to other services; group meetings of peers; group meetings facilitated by someone with training; one-to-one support e.g. counselling; specialist intervention involving mental health services/specialist counselling Other services: Pre-death support Immediate post-death support Home visits Remembrance services Drop-in support Bereavement education for professionals Bereavement sessions in schools and community groups	Wales	**N1:** Team Lead **N2:** Social Worker
Org M	Branch of National Organisation	Bereavement charity	Bereavement support for children and young people (and those caring for them) and adults following all causes of death	Information on grief and sign-posting to other services; one-to-one support e.g. counselling	Northern Ireland	**M2:** Regional Manager **M3:** Volunteer
Org Q	National Organisation	Palliative care charity	Telephone bereavement support for adults following death from a life-limiting illness	Information on grief and sign-posting to other services; one-to-one support e.g. counselling Other services: Online community	UK Wide	**Q1:** Coordinator **Q2:** Volunteer
Org H	Online Organisation	COVID-specific social media group (non-profit)	Online bereavement support for adults following a death from COVID-19, with counsellors moderating the group	Information on grief and sign-posting to other services; online group meetings of peers; online group meetings facilitated by someone with training Other services: Online community support Training for professionals	UK Wide	**H1:** Founder
Org L	Online Organisation	COVID-specific social media group (non-profit)	Online peer-to-peer bereavement support for adults following a death from COVID-19	Information on grief and sign-posting to other services; group meetings of peers Other services: Online community support Memorial services	UK wide, Wales	**L1:** Founder **L2:** Regional Administrator

NGO = non-governmental organisation; NHS = National Health Service (UK).

### Groups with unmet needs

**Figure 2.**
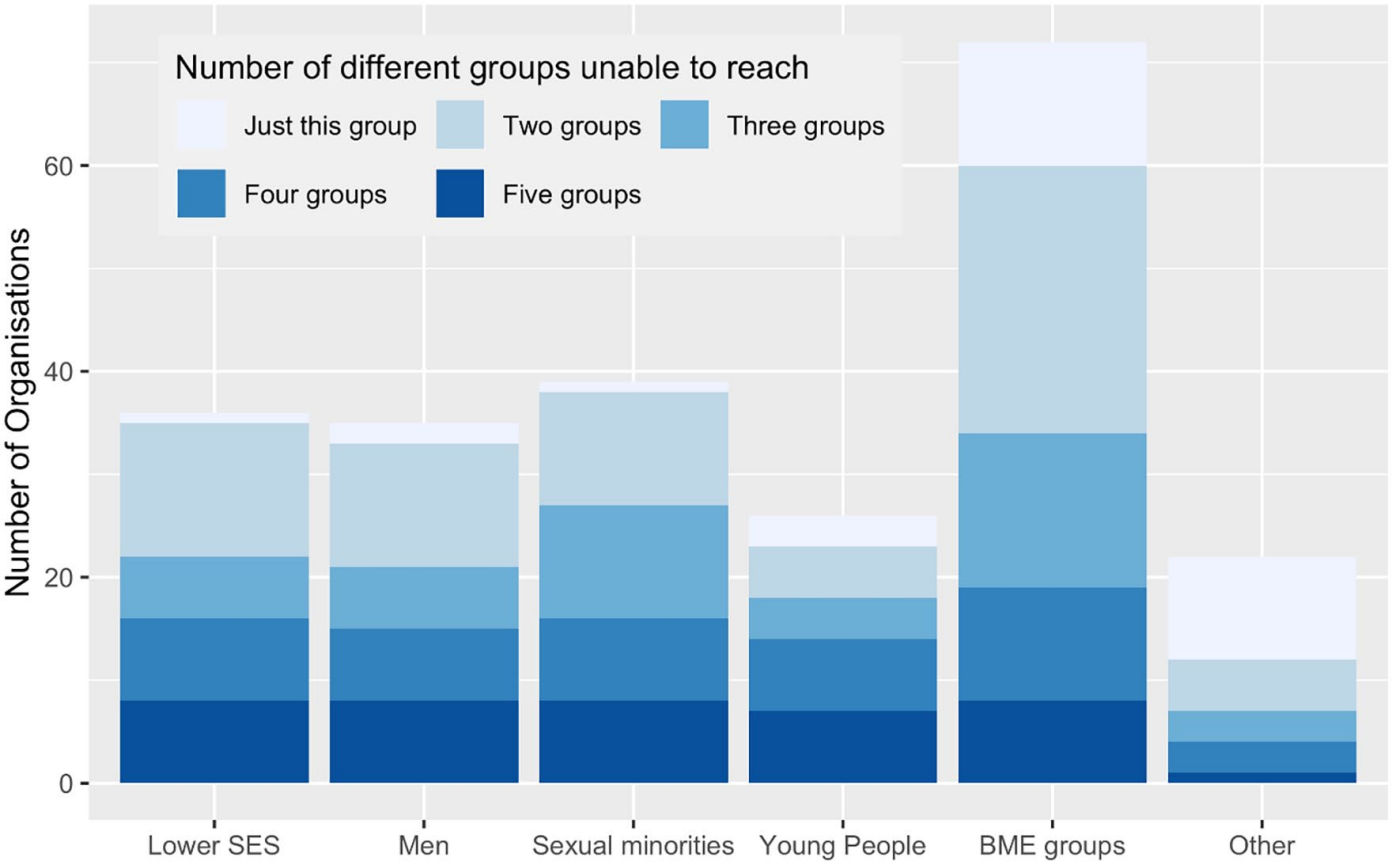
Specific community groups with unmet bereavement support needs not being reached/experiencing access barriers (*N* = 98). SES = socio-economic status; BME = Black and minoritised ethnic. *Note*: Answers to question 15a: If you selected ‘Yes’ [to question 15: *Before COVID-19, do you think there were specific community groups with unmet bereavement support needs that you were not reaching, or who experienced barriers to accessing your service?*)], which groups? Please tick all that apply.

67.3% of services reported that there were groups with unmet needs which were not accessing their services before the pandemic. The most frequently recognised of these was people from minoritised ethnic communities (*n* = 72, 49% of total), followed by sexual minority groups (26.5%), socio-economically deprived communities (24.5%), men (23.8%) and ‘other’ (15%) (including digitally excluded, homeless people, people with learning disabilities, travelling community, non-English speakers, rural communities, physically disabled or with mobility problems). Most organisations that reported being unable to reach certain community groups were not reaching two or more specific groups (71% of those reporting difficulties reaching specific community groups and 48% of the total) (Figure 2). None of the variables used were significant in predicting which organisations were more likely to report being unable to reach specific groups (Supplemental File 3).

The recognition that bereavement support was not equitable was reflected in qualitative data:

*‘one of the big issues that we face as an organisation is actually being able to reach Black and ethnic minority populations, lower socio-economic groups. We really struggle to reach them. You know sadly, well it’s not sad for those people that come to us, but sadly I think we are sort of still quite white, middle-class really.”* (J1, Regional organisation)

*‘I don’t have figures to back this statement up, but from observations, it appears that the client group we are reaching tend to be from a middle class background, they are more educated and have a greater awareness of the support available in the community. We have less referrals from those from a lower socio-economic background.’* (Survey ID122, Branch of national charity/NGO (not bereavement-specific))

### Access by minoritised ethnic communities

50.3% of organisations reported that, in the year before pandemic, <5% of their clients were from minoritised ethnic groups, while 6.8% of services (predominantly London-based) reported that >20% of their clients were from minoritised ethnic groups; 25.2% reported not collecting ethnicity data (Figure 1). 45% of those organisations with less than 5% of their clients from minoritised ethnic groups did not report that those communities had unmet needs for their support.

**Figure 3.**
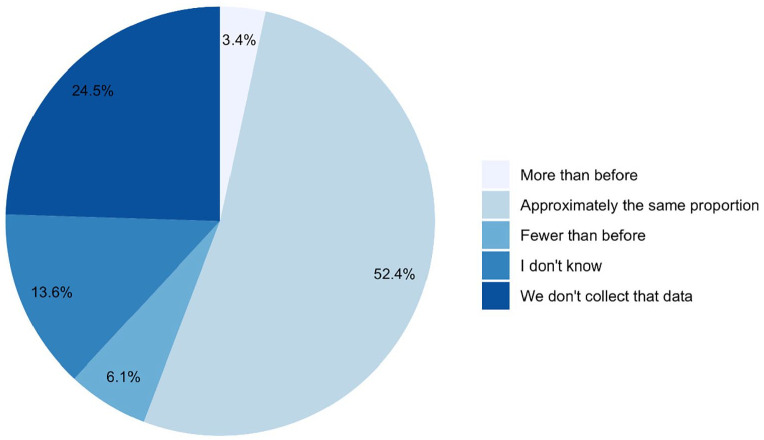
Change in proportion of clients from minoritised ethnic communities since before the COVID-19 pandemic (*n* = 147).

**Figure 4.**
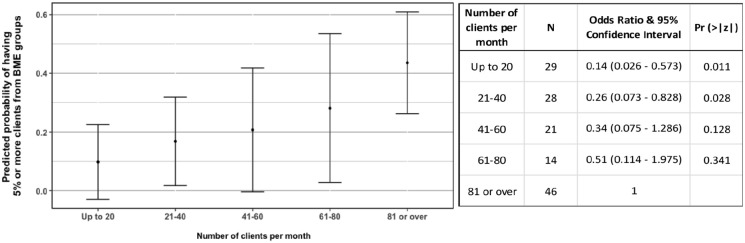
Left – Probabilities of an organisation having 5% or more clients from minoritised ethnic groups in relation to number of clients per month, predicted from the Logistic Regression parameters (Supplemental File 3). The final model which predictions were calculated from also included the variable ‘focus on children and young people’, which was set to ‘yes’, as the best represented category. Right – Table of Odds Ratios and 95% Confidence Intervals for Number of clients per month (reference category: 81 or over).

There was a trend towards an increasing number of clients (i.e. larger organisations) being associated with an increase in the odds of reporting ⩾5% clients from minoritised ethnic groups, but the differences in odds were only significant between the largest organisations and the smallest ones: organisations with ⩾80 clients per month were 3.8 and 7 times more likely to have ⩾5% clients from minoritised ethnic groups compared with organisations with 21 to 40 clients per month and ⩽20 clients per month, respectively (Figure 3 and Supplemental File 3) (Figure 4).

There are apparent regional differences in the proportion of clients from minoritised ethnic groups across organisations (Table 2), but catchment area (UK-wide, nation-specific, county or locally-specific, or other) was not a significant predictor in the analysis. Region of the UK could not be used in the analysis due to very small sample sizes across most regions for services with ⩾5% ethnically minoritised clients (Table 2). There was a possible relationship between organisations supporting primarily children or young clients being more likely to collect information on the ethnicity of their clients (OR = 1.84, 95% CI = 0.938–3.652), but there is a relatively high probability that this result could be due to chance (*p* = 0.077; Supplemental File 3), hence this finding should be considered with caution.

**Table 2.** Contingency table heat-map relating region of the UK served by the organisation with responses to the question ‘In the year before COVID-19, what proportion of your clients were from Black or minority ethnic communities? Please select your closest estimate’. Light-to-dark shading represents increasing numbers of organisations (*n* = 147).

Region of the UK served	Proportion of clients from Black and minoritised ethnic communities							
	Less than 5%	5–10%	11–15%	16–20%	More than 20%	We don’t collect this data	Total which collected this data	Total
UK (all)	5	3	2		2	12	12	24
England (only)					1		1	1
Wales (only)	8	3				3	11	14
Scotland (only)	6	1					7	7
NI (only)	2						2	2
London (greater London)		1		1	5		7	7
South East	10	3	2			11	15	26
East of England	6	1	1			2	8	10
East Midlands	4	1				1	5	6
North East	2	1				1	3	4
North West	10					3	10	13
West Midlands	4		1		2	2	7	9
South West	8	1	1	1			11	11
Yorkshire & the Humber	5	2				2	7	9
East Midlands AND South East	1						1	1
North West AND Wales	1						1	1
Wales AND West Midlands	1						1	1
Yorkshire & Humber AND East Midlands	1						1	1

Light shading = few organisations; dark shading = most organisations

There was variation in how referrals overall had changed during the pandemic, with 35% of services reporting lower numbers compared with before the pandemic and 46% reporting higher numbers (Figure 1). Compared with before the pandemic, 3.4% of services were seeing more people from minoritised ethnic groups, 6.1% were seeing fewer and 38% either didn’t know or didn’t collect this data (Figure 3).

The variation in access by people from minoritised ethnic communities was reflected in qualitative data, for example:

*Massive increase in access by our South Asian and African Caribbean communities – Early access – more people are coming to us within the first 6 months of their bereavement – Covid-19 is the second highest cause of death – 60% increase in access – compared to same 12 months last year.*’ (Survey ID94, Branch of national bereavement charity/NGO)

*We’ve had so many less referrals from ethnic minorities than normal, yeah, and if we’ve had, if someone’s identified themselves from an ethnic minority background, they’ve usually got really good English, whereas before we have had to use our interpreting service or some other way of talking to people.* (A1, Hospice)

### Disproportionate impact and cultural appropriateness

Participants from services supporting minoritised ethnic communities described the multidimensional and acute impact of the COVID-19 pandemic on their clients, in terms of the number and nature of deaths but also the disruption to caring practices:

• *‘We think that the increase in minority groups contacting us is directly related to the numbers of the BAME [Black, Asian and minoritised ethnic] community dying from Covid-19. Very sadly, some of the callers to our service have had almost whole families wiped out from Covid-19.’* (Survey ID44, National bereavement charity/NGO)

• *In terms of the way some families work and the dynamics of them; there’s real emphasis on caring for your own elders and all of that, again, has just been eliminated by the fact that these deaths are often – in fact, the vast majority – in hospital wards and because of the restrictions it’s not possible to go and see your loved one until you get that call to say they’re dying. . . so it’s sudden, it’s unexpected, there isn’t that closure. Afterwards, too, the support that you tend to get after from the wider community visiting your home and offering their consolation and things like that, that’s all absent as well.*’ (B1, Small organisation for minoritised ethnic groups)

This disruption also extended to mourning traditions, as infection control restrictions had a disproportionate impact on communities with strong and important rituals involving cleansing or viewing the deceased’s body or coming together in large groups to support the bereaved:

*From the Muslim perspective. . . there are certain rites when it comes to burial and death. . . It might sound very odd to somebody who doesn’t share those beliefs, but one of the things that is really important is to be part of that burial process, to wash and shroud the deceased loved ones of yours. It’s almost as though there’s a pride, almost, for that individual to give them away to what we believe is the next world, if you like – we believe in the hereafter, life after death – and the closure that that would’ve resulted in has gone away into the ether.’* (B1, Small organisation for minoritised ethnic groups)

*‘BAME [Black, Asian and minoritised ethnic] communities have expressed interruption of spiritual and cultural rituals of marking the passing of a loved one in all diverse communities.’* (Survey ID32, National bereavement charity/NGO)

These particular challenges in bereavement highlighted the importance of cultural appropriateness in bereavement support and the role of religious support and guidance:

*We also offer support from a religious perspective. . . ‘Where is my child now. . .?. . .If I were to visit their grave, would they be able to hear me?’, those sorts of questions are really quite important to grief processes [and]. . . can only really be answered by somebody who has an insight into those things. We don’t always have all the answers. . . but we do have access to imams and resources. . . over time we’ve accumulated a database of commonly-asked questions.*’ (B1, Small organisation for minoritised ethnic groups)

Understanding and accommodating cultural traditions was essential; for example, a participant from a service focused on supporting African Caribbean communities contrasted a family’s poor experiences at a national charity with their own understanding and accommodation of African Caribbean traditions such as 9 Nights:

*I have heard stories, not to pick on [National Organisation], but I have heard stories, family that came to us said they didn’t have a good experience. . . We can go into things you know, we have our references, how we do things. . . we do our wakes, what we call 9 Nights and all the traditional things that we can also include in our support. . . We understand that expression.’* (D1, Small organisation for minoritised ethnic groups)

Finally, specific cultural stressors which need to be understood by bereavement support providers were described; for example, how close community support can sometimes present its own challenges:

*‘In the Muslim community we tend to have very much a community spirit and we do tend to help one another but sometimes that can be quite suffocating . . . it’s really hard to confide in people without making them upset or making them feel more hurt than they already are. . . so you tend to kind of bottle things up. . . [but] you’re still carrying the grief aren’t you.’* (B2, Small organisation for minoritised ethnic groups)

### Effects of the pandemic on access

Participants reported a huge move to online services, which had both positive and negative impacts on accessibility. On the one hand, it reduced waiting times and improved reach and accessibility for groups who might have been excluded by face-to-face services, for example, carers, those in rural communities and Muslim women:

*‘Muslim women tend to go through something called the Iddah period after they lose their husband where, for four months and 10 days, it’s a period of reflection where they tend to stay at home, and for them to access this support is now a possibility.’* (B1, Small organisation for minoritised ethnic groups)

It also benefitted groups who preferred online provision – men and young people were mentioned in this regard, for example

*‘Yes – the remote nature and greater use of social media has helped to engage a younger population.’* (Survey ID119, National bereavement charity/NGO)

With the use of online services, access to support could also be faster and less restricted – for example, by where volunteers were based. However, some people were excluded, particularly people already experiencing disadvantage, for example, due to illiteracy or a lack of access to technology:

*‘Having these session via Zoom will exclude some people but when I’m asking the triage team on our helpline what percentage of people does it exclude, they would say about 10-15%, so the majority of people [can access it], even our older community. . . It tends to be people with maybe very serious mental health conditions or on the peripheries of society or who are digitally excluded obviously can’t attend*. ‘ (C1, Branch of Large National Organisation)

*‘We’ve got an illiteracy rate of something like 20 percent in [area] so our client group are already disadvantaged by poor reading and writing skills irrespective of their technological skills.’* (N1, Hospice)

Other groups reported to find online support difficult or impossible included young children, those who required text speak or translation services, and parents and carers with childcare responsibilities.

Participants described other ways the pandemic had affected services’ ability to meet the needs of diverse groups. First, in the context of uncertainty and lack of resources, outreach activities had sometimes been deprioritized or impossible:

*‘However, myself and other colleagues used to go out and promote services face to face to BAME [Black, Asian or minoritised ethnic] groups and obviously this hasn’t happened.*’ (Survey ID2, Hospice)

*‘We have always had our hard-to-reach communities and I think they have potentially become harder to reach because they closed off during COVID, so we would want to start to engage that work again. . . We are just doing a piece of work around palliative care and learning disabilities and we would like to look at homelessness and our travelling community. . . there is lots of scope to re-invigorate work that was already in place that got pulled.*’ (A2, Hospice)

Second, the lack of capacity in mental health services had knock-on effects, with a participant commenting on the lack of follow up from specialist ‘crisis’ teams:

*‘There have been struggles or perhaps capacity issues with people when for instance we’ve contacted Crisis teams and they haven’t necessarily followed up on individuals. So I get the sense. . . that it’s been quite stretched or difficult in some areas.*’ (Q1, Large National Organisation)

### Positive interventions

Service providers described positive interventions they had implemented or wanted to implement to try to reduce inequity of access. A fundamental step was capturing clients’ demographic data to understand who was and wasn’t accessing the service:

*‘We do not capture data on sexuality, race etc. for all of our clients at the beginning of accessing our support, this is only captured at the end and is optional (so we don’t have data that represents the majority of people accessing our support). We are looking at better ways to be capturing this data so it gives a more accurate representation.*’ (Survey ID73, Branch of a national charity/NGO)

Through collaboration with community groups and other organisations as well as GPs, services aimed to improve referral pathways, signposting, outreach and advertising to specific communities:

*‘We have partnered with [Black women’s group] and this has developed engagement from BAME [Black, Asian or minoritised ethnic] communities.*’ (Survey ID139, National bereavement charity/NGO)

*‘It has been positive to work with other local charities and organisations. Connections not only local, but Twitter/Facebook have enabled wider contact, sharing of knowledge, reduction in barriers’.* (Survey ID80, Community-led peer support)

Attending to language and representation was seen a key way of improving access:

‘*On our triage team, one of my colleagues, Imrat, she speaks Punjabi and Urdu, we’ve recruited a lot more volunteers as well that speak various languages, so it’s making us more accessible, more diverse, which is amazing. Anita, who also answers the triage line, she’s from the West Indies, she’s got a very distinct accent, and do you know what, it makes callers feel very, very comfortable. . . just to have a diverse range of people answering the triage line is incredible.*’ (C2, Branch of Large National Organisation; pseudonyms used)

Some organisations were engaged in projects to try to reach marginalised groups, had introduced new services to target perceived gaps in their provision and/or reported supporting other professionals (e.g. community workers) to provide bereavement support themselves. Other activities included engaging an external organisation to look at equality and diversity across an organisation, and designing and implementing an organisational strategy focussed on inclusivity, diversity and outreach; for example:

*‘Equality and diversity is a big theme in [National Organisation] at the minute, and we’ve actually engaged an organisation to have a look at all our stuff and see how, from an objective point of view, how it all looked in terms of equality and diversity . . . I have linked in with groups. . . that support LGBTQ communities. . . about. . . what makes us as an organisation approachable to your clients. . . they need to know who they’re going to for support isn’t going to be shocked or uncomfortable with a same sex relationship. So, for us it’s about, how do we promote [National Organisation] as an organisation that is friendly and supportive for all, race, sex, religion, whatever?*’ (M2, Branch of national organisation)

## Discussion

### Main findings

Two thirds of the UK voluntary and community sector bereavement services that participated in this survey recognised that there were population groups which could benefit from their services, but do not access them. People from minoritised ethnic groups were most frequently recognised in this regard, followed by sexual minority groups, people with lower socio-economic status and men. During the pandemic, on average, proportions of ethnically minoritised clients did not increase despite the disproportionate multidimensional impacts of the pandemic on these communities – in terms of mortality rate and care and mourning practices, but also compounding factors such as social and economic inequity, racism and discrimination.

### What this study adds

One approach to widening access was to expand advertising and focus on outreach, for example via other community organisations or groups. Proactive advertising via local community networks and organisations was an important feature of the immediate disaster response to the 911 attacks in New York, which achieved high uptake of counselling support among minoritised ethnic groups.^35,36^ Similarly, interventions which focus on language and visibility, which we also identified, can help encourage engagement with formal bereavement support.^5,35^

However, previous studies have highlighted cultural inappropriateness in bereavement support^37,38^ and the limitations of a ‘blanket’ approach which does not address individual needs.^39^ If services are, albeit unintentionally, inappropriate, insensitive or biased, then raising awareness of services among disadvantaged communities, providing linguistic access or ensuring a diverse workforce will not, on its own, create equity. Appointment of bilingual health-care workers can help bring about better family support both before and after the death,^40^ yet it is their awareness of cultural proprieties around death and mourning which is likely crucial to their success.

More extensive interventions to widen access to bereavement support which we identified therefore examined how organisational structures and approaches to care could exclude diverse groups from engaging with or benefitting from bereavement services and adapted services accordingly. These kinds of interventions are often based on consultation or co-production with disadvantaged groups, prioritise cultural competence and service adaptation, and may involve collaboration with other organisations with specific cultural, faith, legal or financial expertise to help meet group-specific support needs. Systematic reviews have established the importance of cultural knowledge and sensitivity in bereavement support following mass-bereavement events^35^ and in palliative care.^5^ A recent survey of mental health services for minoritised ethnic communities in the UK similarly identified a need for mainstream therapists and service providers to have quality-assured cultural competency training.^12^ The survey also identified increased demand for bereavement support provided by organisations led by people from minoritised ethnic commmunities during the pandemic,^12^ highlighting the importance of supporting community organisations representative of and trusted by the populations which they serve.

While further research into the formal and informal bereavement support needs of specific minoritised communities is undoubtedly needed, in the absence of this evidence, assumptions about community norms, such as ‘they look after their own’, should be avoided.^41^ Such assumptions can prevent services from critical reflection on how they operate, further increasing inequities in support. Instead of dichotomising professional and community bereavement support, we need to attend to and invest in all tiers of the public health model^3,42^: informal support, information and sign-posting to other forms of support for all bereaved people; peer-to-peer and community group support for people who need a more formal opportunity to review their experience of loss, but not necessarily with professionals; and specialist interventions such as mental health and psychological support services for those people who require them. Equity must be a priority at all three levels. Given that social isolation and loneliness are often higher in socio-economically disadvantaged populations,^43^ that bereaved people report significant challenges accessing support from family and friends^25^ and that many people do not feel comfortable responding to or supporting someone who has been bereaved,^11,44^ efforts to improve all communities’ grief literacy are essential.^45^ Bereavement services, if mindful of the known dangers of over-professionalisation,^46^ can play a key role in supporting these efforts, working as equal partners with communities and voluntary and community sector organisations.

### Limitations

We did not collect detailed data on the range of groups not accessing services or how these might have changed during the pandemic. Our decision to focus on minoritised ethnic groups was informed by evidence of the impact of the pandemic at the time of the survey and pragmatic concerns regarding participant burden. Research into access to bereavement services by other disadvantaged groups is crucial given what is now known regarding the pandemic’s impact.^47^ Convenience sampling might have resulted in less burdened or more engaged services completing the survey. It is not known precisely how many voluntary and community sector bereavement services there are in the UK; a 2020 analysis of services registered on a national directory identified 822 entries,^30^ however this is likely to include services outside the sector and services no longer operating. For practical reasons the survey considered marginalised groups as separate entities rather than intersecting in complex ways; study interviews with bereaved people will explore access and intersectionality in more depth. Given diversity in types of catchment area, it was not possible to conduct a detailed analysis of proportions of ethnically minoritised clients in services serving distinct regions of the UK and compare these with local population characteristics.

### Implications and recommendations

On the basis of study findings, we recommend that:

- Collection of client data on all key protected characteristics^6,48^ and outcomes of support becomes routine in all bereavement services, to enable a better understanding of access, ‘reach’ and effectiveness, to help ensure equity and meet the needs of the whole population. A quarter of participating services currently do not have accurate data relating to ethnicity.
- Services assess unmet needs for formal bereavement support in the local community, recognising that not everyone will need professional support but that appropriate support should be offered and available to anyone who might benefit. Client data can then be compared with local population characteristics and needs assessment, based on catchment area and target population. Within an organisation, open discussion of this data may help create internal motivation^49^ to change practice and improve equity.
- Because a ‘one size fits all’ approach will never achieve equity, service providers must ask sometimes difficult and uncomfortable questions about the nature of their service, and build on basic interventions such as outreach, language accessibility and diversity of representation to consider how organisational structures and assumptions could preclude beneficial support.
- Implicit bias, anti-discrimination and cultural competency training should be routine for bereavement providers – and the mental health services they refer to and which signpost to them.
- Local knowledge of community assets, collaboration with other community-based organisations, and meaningful co-production should be standard approaches in the design and delivery of bereavement services. This is complex and potentially challenging work, and will require investment in resources to support community engagement and capacity building, including training to build practitioner knowledge and skills.
- Since online services have drawbacks as well as benefits in terms of accessibility, with the most disadvantaged often the most likely to be excluded, research is needed to further understand the acceptability and outcomes of online support in different groups.
- Financial resources and support are provided to community organisations working with minoritised groups, strengthening the bereavement support that they are able to provide to the communities they serve. Bereavement services can help advise and support other community stakeholders and bolster, rather than replace, the care provided by social networks.
- Research is conducted to identify best practice interventions to reduce inequity in access to bereavement support.

## Supplemental Material

sj-docx-1-pmj-10.1177_02692163221133665 – Supplemental material for ‘Sadly I think we are sort of still quite white, middle-class really’ – Inequities in access to bereavement support: Findings from a mixed methods study Click here for additional data file. Supplemental material, sj-docx-1-pmj-10.1177_02692163221133665 for ‘Sadly I think we are sort of still quite white, middle-class really’ – Inequities in access to bereavement support: Findings from a mixed methods study by Lucy E Selman, Eileen Sutton, Renata Medeiros Mirra, Tracey Stone, Emma Gilbert, Yansie Rolston, Karl Murray, Mirella Longo, Kathy Seddon, Alison Penny, Catriona R Mayland, Donna Wakefield, Anthony Byrne and Emily Harrop in Palliative Medicine

sj-pdf-2-pmj-10.1177_02692163221133665 – Supplemental material for ‘Sadly I think we are sort of still quite white, middle-class really’ – Inequities in access to bereavement support: Findings from a mixed methods study Click here for additional data file. Supplemental material, sj-pdf-2-pmj-10.1177_02692163221133665 for ‘Sadly I think we are sort of still quite white, middle-class really’ – Inequities in access to bereavement support: Findings from a mixed methods study by Lucy E Selman, Eileen Sutton, Renata Medeiros Mirra, Tracey Stone, Emma Gilbert, Yansie Rolston, Karl Murray, Mirella Longo, Kathy Seddon, Alison Penny, Catriona R Mayland, Donna Wakefield, Anthony Byrne and Emily Harrop in Palliative Medicine

sj-pdf-3-pmj-10.1177_02692163221133665 – Supplemental material for ‘Sadly I think we are sort of still quite white, middle-class really’ – Inequities in access to bereavement support: Findings from a mixed methods study Click here for additional data file. Supplemental material, sj-pdf-3-pmj-10.1177_02692163221133665 for ‘Sadly I think we are sort of still quite white, middle-class really’ – Inequities in access to bereavement support: Findings from a mixed methods study by Lucy E Selman, Eileen Sutton, Renata Medeiros Mirra, Tracey Stone, Emma Gilbert, Yansie Rolston, Karl Murray, Mirella Longo, Kathy Seddon, Alison Penny, Catriona R Mayland, Donna Wakefield, Anthony Byrne and Emily Harrop in Palliative Medicine
