# Supplementary material for: ‘Sadly I think we are sort of still quite white, middle-class really’ – Inequities in access to bereavement support: Findings from a mixed methods study
Source: Palliat Med. 2022 Nov 6;37(4):586–601. doi: 10.1177/02692163221133665 (PMC10074747; doi:10.1177/02692163221133665)
Supplement: sj-pdf-3-pmj-10.1177_02692163221133665 – Supplemental material for ‘Sadly I think we are sort of still quite white, middle-class really’ – Inequities in access to bereavement support: Findings from a mixed methods study [file sj-pdf-3-pmj-10.1177_02692163221133665.pdf]

Table I. Results of logistic regression for there being specific community groups with unmet bereavement support needs not being reached/experiencing barriers to accessing your service (yes or no)

	Full model					Reduced Model				
	Df	Deviance	AIC	LRT	Pr(>Chi)	Df	Deviance	AIC	LRT	Pr(>Chi)
Intercept	157.84	197.84				184.66	188.66			
Catchment area	3	158.98	192.98	1.143	0.767					
Organisation type	6	163.13	191.13	5.297	0.506					
Focus on children & young people	1	157.84	195.84	0.009	0.926					
Focus on adults	1	158	196	0.161	0.688					
Focus on other age groups	1	157.86	195.86	0.027	0.869					
Restricted to specific groups	1	158.4	196.4	0.563	0.453	1	185.72	187.72	1.063	0.303
Specialise in age of deceased	1	158.83	196.83	0.996	0.318					
Support for all causes of death	1	159.16	197.16	1.32	0.251					
Number of clients	4	163.97	195.97	6.136	0.189					

Table II. Results of logistic regression for proportion of clients from minoritised ethnic groups (<5% or ≥5%)

	Full Model:					Reduced Model:				
	Df	Deviance	AIC	LRT	Pr(>Chi)	Df	Deviance	AIC	LRT	Pr(>Chi)
Intercept	109.05	149.04				120.81	132.81			
Catchment area	3	110.29	144.29	1.244	0.743					
Organisation type	6	115.95	143.95	6.905	0.330					
Focus on children & young people	1	110.92	148.92	1.879	0.171	1	124.29	134.29	3.479	0.062
Focus on adults	1	110.27	148.27	1.225	0.268					
Focus on other age groups	1	109.36	147.36	0.315	0.575					
Restricted to specific groups	1	109.33	147.33	0.284	0.594					
Specialise in age of deceased	1	109.07	147.07	0.022	0.883					
Support for all causes of death	1	109.13	147.13	0.086	0.770					
Number of clients	4	115.51	147.51	6.468	0.167	4	130.52	134.52	9.701	0.046

Table III. Results of logistic regression for collection of ethnicity data (yes/no)

	Full Model:					Reduced Model:				
	Df	Deviance	AIC	LRT	Pr(>Chi)	Df	Deviance	AIC	LRT	Pr(>Chi)
Intercept	153.38	193.38				192.23	196.23			
Catchment area	3	157.97	191.97	4.5914	0.20428					
Organisation type	6	162.41	190.41	9.033	0.17173					
Focus on children & young people	1	156.17	194.17	2.7919	0.09474	1	195.37	197.37	3.145	0.077
Focus on adults	1	154.54	192.54	1.1628	0.28088					
Focus on other age groups	1	153.96	191.96	0.5829	0.44519					
Restricted to specific groups	1	155.33	193.33	1.9448	0.16315					
Specialise in age of deceased	1	153.68	191.68	0.2979	0.5852					
Support for all causes of death	1	153.77	191.77	0.3898	0.5324					
Number of clients	4	159.46	191.46	6.0754	0.19359					
